# Prolongation of Relaxation Time in Extraocular Muscles With Brain Derived Neurotrophic Factor in Adult Rabbit

**DOI:** 10.1167/iovs.16-19679

**Published:** 2016-10

**Authors:** Krysta R. Nelson, Shanlee M. Stevens, Linda K. McLoon

**Affiliations:** Department of Ophthalmology and Visual Neurosciences, University of Minnesota, Minneapolis, Minnesota, United States

**Keywords:** brain derived neurotrophic factor, extraocular muscles, muscle force, muscle physiology, slow myosin

## Abstract

**Purpose:**

We tested the hypothesis that short-term treatment with brain derived neurotrophic factor (BDNF) would alter the contractile characteristics of rabbit extraocular muscle (EOM).

**Methods:**

One week after injections of BDNF in adult rabbit superior rectus muscles, twitch properties were determined in treated and control muscles in vitro. Muscles were also examined for changes in mean cross-sectional areas, neuromuscular junction size, and percent of myofibers expressing specific myosin heavy chain isoforms, and sarcoendoplasmic reticulum calcium ATPases (SERCA) 1 and 2.

**Results:**

Brain derived neurotrophic factor–treated muscles had prolonged relaxation times compared with control muscles. Time to 50% relaxation, time to 100% relaxation, and maximum rate of relaxation were increased by 24%, 27%, and 25%, respectively. No significant differences were seen in time to peak force, twitch force, or maximum rate of contraction. Brain derived neurotrophic factor treatment significantly increased mean cross-sectional areas of slow twitch and tonic myofibers, with increased areas ranging from 54% to 146%. Brain derived neurotrophic factor also resulted in an increased percentage of slow twitch myofibers in the orbital layers, ranging from 54% to 77%, and slow-tonic myofibers, ranging from 44% to 62%. No significant changes were seen SERCA1 or 2 expression or in neuromuscular junction size.

**Conclusions:**

Short-term treatment with BDNF significantly prolonged the duration and rate of relaxation time and increased expression of both slow-twitch and slow-tonic myosin-expressing myofibers without changes in neuromuscular junctions or SERCA expression. The changes induced by BDNF treatment might have potential therapeutic value in dampening/reducing uncontrolled eye oscillations in nystagmus.

Neurotrophic factors have a broad range of functions in both developing and mature nervous system and muscles. Brain derived neurotrophic factor (BDNF) is one such factor. It has a myriad of functions, which includes control of neuronal survival, axon growth, and plasticity,^1^ and is a key regulator of synapse development and plasticity.^2^ Brain derived neurotrophic factor is expressed during skeletal muscle development where it serves as a retrogradely transported survival factor for motor neurons.^3,4^ Specifically in the developing oculomotor system, BDNF was shown to be target derived, where application to the eye muscles was shown to be retrogradely transported to the appropriate motor neurons.^5^ Its expression is downregulated in adult limb skeletal muscle, where it is expressed mainly in satellite cells and colocalized with the low affinity receptor p75.^6^ Its expression patterns and its potential functional role in adult extraocular muscles (EOM) are not understood. We recently showed that both BDNF and tropomyosin receptor kinase B (trkB), the receptor for BDNF, are expressed in adult EOM.^7^ This study sought to determine if exogenous treatment with BDNF could alter contraction or relaxation rates in adult rabbit EOM.

The neurotrophic and muscle growth factors that control normal EOM structure and function are not well characterized. Additionally, it is not known how perturbations in the levels of these neurotrophic and growth factors might play a role in eye movement disorders during normal maturation of the oculomotor control system. Recent studies demonstrated that insulin-like growth factor-1 (IGF-1) applied unilaterally over a 3 month time-period to infant monkey medial rectus muscle resulted in development of strabismus,^8^ while BDNF treatment did not.^7^ However, analysis of the effects of sustained release of BDNF showed that there was a significant effect on the myofibers that expressed the slow myosin heavy chain isoform (MyHC). Not only were these BDNF-treated myofibers significantly larger than age-matched control slow myofibers after the 3 months of continuous BDNF exposure, there was also an increased number of slow-positive myofibers in these BDNF-treated infant extraocular muscles. This suggests that the BDNF had an effect on fiber type switching.^7^ Additionally, the neuromuscular junctions on the slow myofibers were also proportionally enlarged. These studies support the view that growth factors can significantly influence fiber type characteristics and function.

Examination of extraocular muscles collected from children with infantile nystagmus syndrome (INS) at the time of surgery showed that these muscles contained significantly smaller neuromuscular junctions compared with normal age-matched control extraocular muscles.^9^ In addition, the extraocular muscles from the INS subjects had both larger and increased numbers of slow MyHC-positive myofibers. Due to the nature of muscles collected from INS subjects undergoing surgical procedures, we cannot assess whether the differences between their muscles and controls were primary or secondary. If secondary, we hypothesize that these changes could be an attempt to stabilize or otherwise minimize the uncontrolled oscillatory movements of the eyes. Supporting evidence for the potential role of BDNF in stabilization of oscillatory movements comes from the Pastor laboratory, where application of BDNF after an abducens nerve section in adult cats resulted in restoration of the tonic portion of motor neuron firing, in contrast with neurotrophin-3, which restored the phasic firing.^10^ This supports the view that different neurotrophic factors control different aspects of EOM structure and function. Thus, we specifically examined the effect of BDNF treatment on the contractile properties of adult rabbit EOM using in vitro muscle force assessment and asked whether BDNF would alter the contractile profile of the treated muscles. We also assessed if these changes correlated with changes in slow and/or slow tonic MyHC expression. If slow MyHC-expressing myofibers were elevated in number or size, we hypothesize that these changes in fiber type would be expected to produce a slower contractile profile compared with fast MyHC-expressing myofibers. In addition, we examined changes in fast, developmental, and neonatal MyHC expression and as well as the expression patterns for the sarcoendoplasmic reticulum calcium ATPases (SERCA) 1 and 2. The SERCAs are known to play an important role in the regulation of fiber relaxation rate^11^; thus, we examined the EOM myofibers for potential changes in expression levels of these two proteins. Length and area of neuromuscular junctions were also determined, as previous studies suggested that neuromuscular junction size may change in response to BDNF treatment.^7^

## Methods

Adult New Zealand White rabbits were obtained from Bakkom Rabbitry (Viroqua, WI, USA) and housed in the animal facility at the University of Minnesota. All experiments were approved by the Institutional Animal Care and Use Committee at the University of Minnesota, and complied with the guidelines issued by the National Institutes of Health and ARVO.

All rabbits were anesthetized with ketamine (100 mg/ml):xylazine (20 mg/ml) at a ratio of 4:3, injected intramuscularly. For physiological analysis, eight rabbits received an intramuscular injection of 1 μg BDNF (R&D Systems, Minneapolis, MN, USA) in a volume of 100 μl sterile saline directly into a superior rectus muscle. The contralateral side was injected with an equal volume of sterile isotonic saline. Rabbits received injections on three sequential days. A second set of eight age-matched control rabbits was used as a noninjected control.

One week after the first injection, the superior rectus muscles were removed for analysis using our in vitro muscle physiology system and our standard protocol (Aurora Scientific, Aurora, Ontario, Canada).^12^ To summarize, the rabbits were deeply anesthetized with ketamine and xylazine and were euthanized by exsanguination and bilateral thoracotomy. The superior rectus muscles were dissected from the sclera to the apex of the orbit and placed immediately into oxygenated Ringers solution at 32°C. Both ends were tied with 4.0 silk suture, and loops at each end were attached to a stationary bar and to a lever arm that was connected to a force transducer. The muscles were then immediately submerged in an oxygenated Ringers bath. Muscles were globally stimulated by two electrodes, and force was measured in grams. After determination of optimal preload, followed by a 15-minute rest, the muscles were stimulated at 10, 20, 40, 100, 150, and 200 Hz, allowing 2 minutes of rest between each stimulation. Stimulation of the superior rectus muscles from the right and left sides was alternated. Peak force was defined as the maximal force at any specific frequency. Twitch profiles were analyzed as time to 50% relaxation (half relaxation time) in seconds, time to 100% relaxation in seconds, maximum force generated in grams, time to maximum force in seconds, peak rate of contraction in grams/second (dF/dt), and peak rate of relaxation in grams/second (dF/dt). After 2 minutes, a fatigue protocol was followed, where a tetanic stimulus was delivered every 2 seconds composed of a train of stimulations for 1 second at 150 Hz. Muscles were stimulated for 600 seconds, and time of 50% reduction in maximum tetanic force was calculated in seconds.

A separate series of five adult New Zealand White rabbits received injections on 3 consecutive days of 1 μg BDNF in 100-μl sterile saline in one superior rectus and an injection of saline only in the contralateral superior rectus. One week after the first injection, the animals were euthanized, and both superior rectus muscles were dissected from sclera to apex of the orbit, frozen in methylbutane, and sectioned at 10 μm. In addition, a series of superior rectus muscles from five control rabbits that had received no treatments were also sectioned at 10 μm and processed similarly to the experimental tissues.

Immunohistochemical staining for slow-twitch (1:40; Vector Labs, Burlingame, CA, USA), slow-tonic (1:50; S46-c; Developmental Studies Hybridoma Bank, Iowa City, IA, USA), fast (1:40; Vector Labs), developmental (1:20; Vector Labs), and neonatal (1:40; Vector Labs) MyHC isoforms was performed. Additional sections were immunostained for SERCA1 and 2 (1:2000; Affinity Bioreagents, Golden, CO, USA). Sections were incubated in primary antibody for 1 hour, rinsed in PBS, incubated using the Vectastain Kit (Vector Labs) following package directions, and colorized using diaminobenzidine and hydrogen peroxide. Slides were dehydrated and coverslipped. For the fluorescent identification of neuromuscular junctions, sections were immunostained for the slow twitch MyHC isoform and then incubated with a secondary antibody conjugated to Dylight 405 (1:100: Jackson ImmunoRes Labs, West Grove, PA, USA), followed by incubation with α-bungarotoxin conjugated to Alexa Fluor 488 (1:500; ThermoFisher Scientific, Waltham, MA, USA). Slides were visualized using a Leica DM4000 microscope (Buffalo Grove, IL, USA).

Mean cross-sectional area and percentage of myofibers positive for each of the five MyHC isoforms and for SERCA1 and 2 were determined for both the orbital and global layers in the midregion and toward the tendon region, ensuring that both layers were present, for the BDNF-treated superior rectus muscle, the contralateral superior rectus muscle, and naïve control superior rectus muscles using Bioquant Image Analysis software (Bioquant, Nashville, TN, USA). A minimum of 300 myofibers were analyzed in both the orbital and global layers of each superior rectus muscle in both the midregion and toward the scleral tendon end where the orbital layer was still present. Three to five sections were analyzed per region and per muscle, and the percent and mean cross-sectional areas were averaged. For myofibers expressing fast, slow-twitch, slow-tonic, developmental, and neonatal MyHC isoforms both percent positive and mean cross-sectional areas were determined. Slow tonic MyHC immunostaining in rabbit extraocular muscles was similar to that in the published literature for other species.^13^ For myofibers expressing SERCA1 and 2, only the percentage of positive myofibers was determined. In addition, length and area of neuromuscular junctions were analyzed in both the midregion and toward the tendon region for mean length, mean area, mean length as a percent of myofiber perimeter, and mean area as a percent of myofiber area. All analyses were performed using the Bioquant Image Analysis System (Bioquant Image Analysis Corp.). For the final analyses, the averages from each rabbit were used to calculate the mean ± SEM.

Data between the three groups of muscles were analyzed for significance using an ANOVA followed by Dunnett's or Tukey's multiple comparison tests. Statistical significance was defined as *P* less than 0.05.

## Results

**Figure 1:**
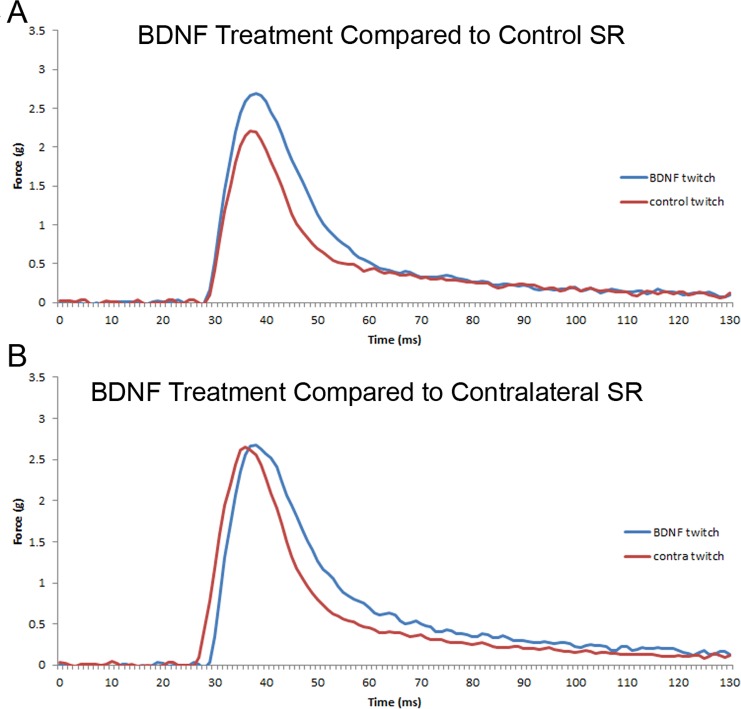
Single-twitch contractions generated at 10-Hz stimulation frequency graphed as change in force over time. (**A**) Naïve control single twitch (*red*) compared with a single twitch from a muscle treated 1 week earlier with BDNF (*blue*). (**B**) A single twitch from a muscle treated 1 week earlier with BDNF (*blue*) compared with a single twitch from the superior rectus muscle of the saline injected contralateral side (*red*). Time to twitch was adjusted for figure.

**Figure 2:**
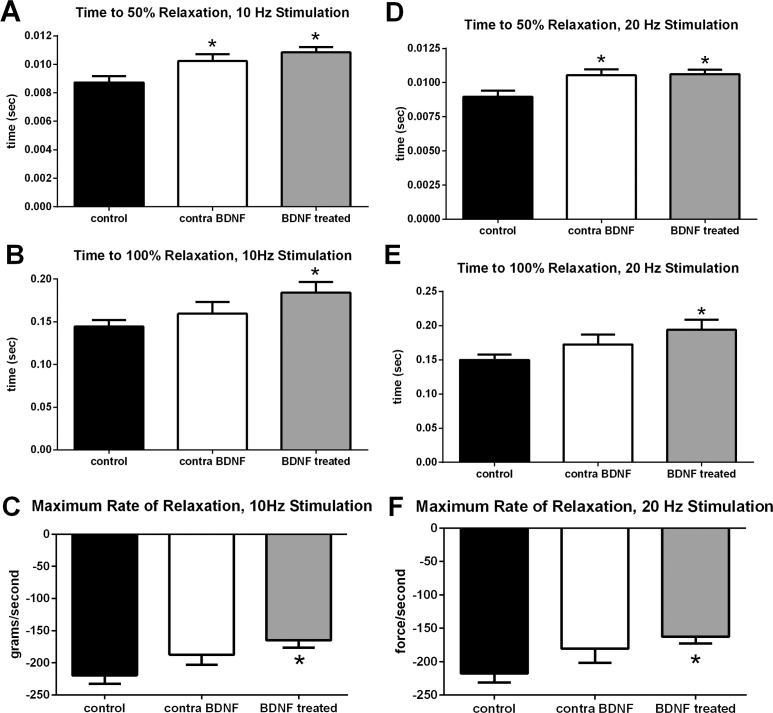
Mean values after 10- Hz stimulation for (**A**) time to 50% relaxation, (**B**) time to 100% relaxation, and (**C**) maximum rate of relaxation from naïve control superior rectus muscles (*black bar*), the superior rectus muscle on the contralateral side from the treated muscle (contra; *white bar*), and the BDNF-treated superior rectus muscles (*light gray bar*). Mean values after 20-Hz stimulation for (**D**) time to 50% relaxation, (**E**) time to 100% relaxation, and (**F**) maximum rate of relaxation from untreated control superior rectus muscles, BDNF-treated superior rectus muscles, and from the superior rectus muscle on the contralateral side from the treated muscle. *Significant difference from naïve control measurements.

Superior rectus muscles were examined for the effect of intramuscular injection of BDNF on contractile properties. After twitch stimulation at 10 Hz, BDNF injected into a superior rectus muscle unilaterally resulted in a prolongation in relaxation times (Figs. 1A, 2). Specifically, BDNF-treated superior rectus muscles showed a significant 24.23% prolongation in 50% relaxation time, with a mean of 11 ± 0.4 ms one week after BDNF treatment compared with naïve control muscles at 8.7 ± 0.4 ms (Fig. 2A). It is interesting to point out that the superior rectus contralateral to the injected muscle also showed a significant 17.13% prolongation in 50% relaxation time compared with the control muscles, at 10.23 ± 0.5 ms (Figs. 1B, 2). Adaptation of the yoked extraocular muscles is a common finding after surgical or drug manipulation of extraocular muscles,^7,14,15^ so it was not surprising to see. After BDNF treatment, time to 100% relaxation was prolonged 27.58% compared with untreated naïve control muscles, with relaxation times of 184 ± 3 ms compared with controls at 144.3 ± 8 ms (Fig. 2B). In the superior rectus muscles contralateral to the BDNF-treated muscles, relaxation times were not significantly different from naïve control values or from the BDNF-treated muscles. As would be expected, the maximum rate of relaxation after BDNF treatment was prolonged by 24.9% compared with the untreated naïve controls, at −165.0 ± 11.53 g/sec (*df/dt*) compared with the control rate of −219.7 ± 13.14 g/sec (Fig. 2C). The contralateral superior rectus had a rate intermediate between the BDNF-treated and the naïve control superior rectus muscles, at −187.5 ± 15.27 g/sec (*df/dt*), which was not significantly different from either control or BDNF-treated muscles. No other differences were seen in the contralateral superior rectus muscle twitch parameters. Similar effects on prolongation of time to 50% relaxation, time to 100% relaxation, and maximum rate of relaxation were seen after the 20-Hz stimulation in the BDNF-treated superior rectus muscles compared with the naïve control rabbits (Figs. 2D–F). No significant differences were seen in any of these three measurements after stimulation at 40, 100, 150, or 200 Hz (data not shown).

**Figure 3:**
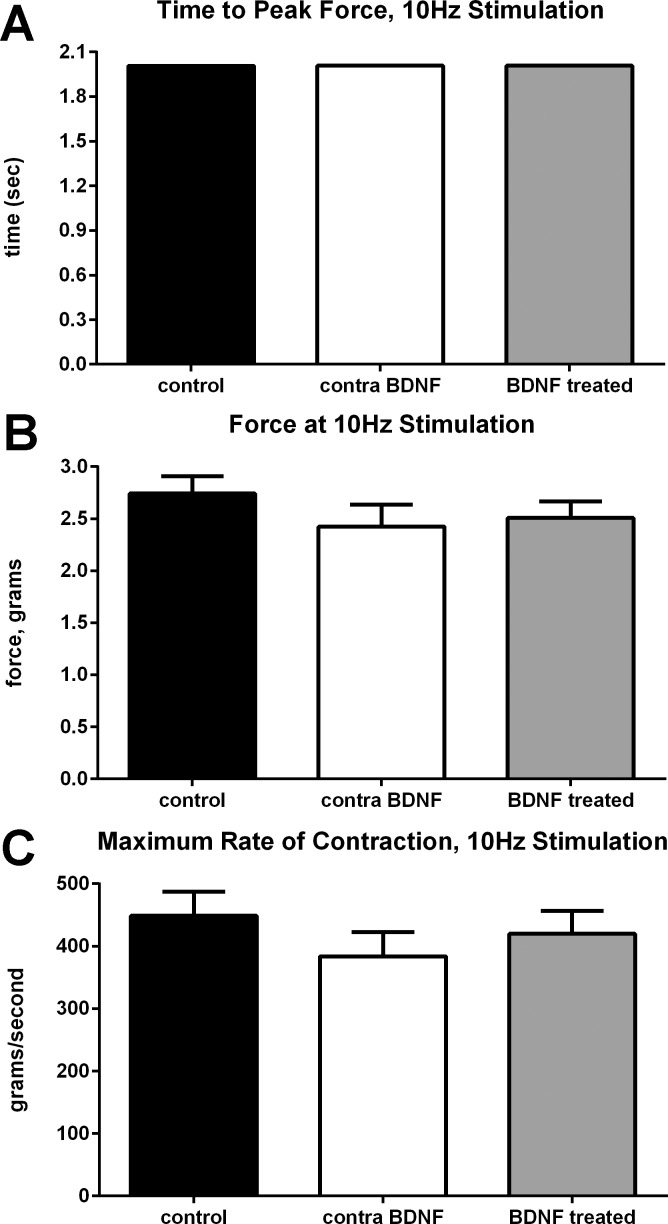
Mean values for (**A**) time to peak force, (**B**) twitch force, and (**C**) maximum rate of contraction from naïve control superior rectus muscles (*black bar*), the superior rectus muscle on the contralateral side from the treated muscle (contra; *white bar*), and the BDNF-treated superior rectus muscles (*light gray bar*). None of the values were significantly different.

In contrast, there were no significant differences between naïve control, BDNF-treated superior rectus muscles, and the muscles contralateral to BDNF treatment in time to peak force (Fig. 3A), overall maximal twitch force (Fig. 3B), or in the maximum rate of contraction (Fig. 3C). Similarly no changes were seen in any of these parameters after 20, 40, 100, 150, or 200 Hz stimulation frequencies. No significant difference was seen in fatigability, as measured by time to 50% of the maximum tetanic amplitude (data not shown).

**Figure 4:**
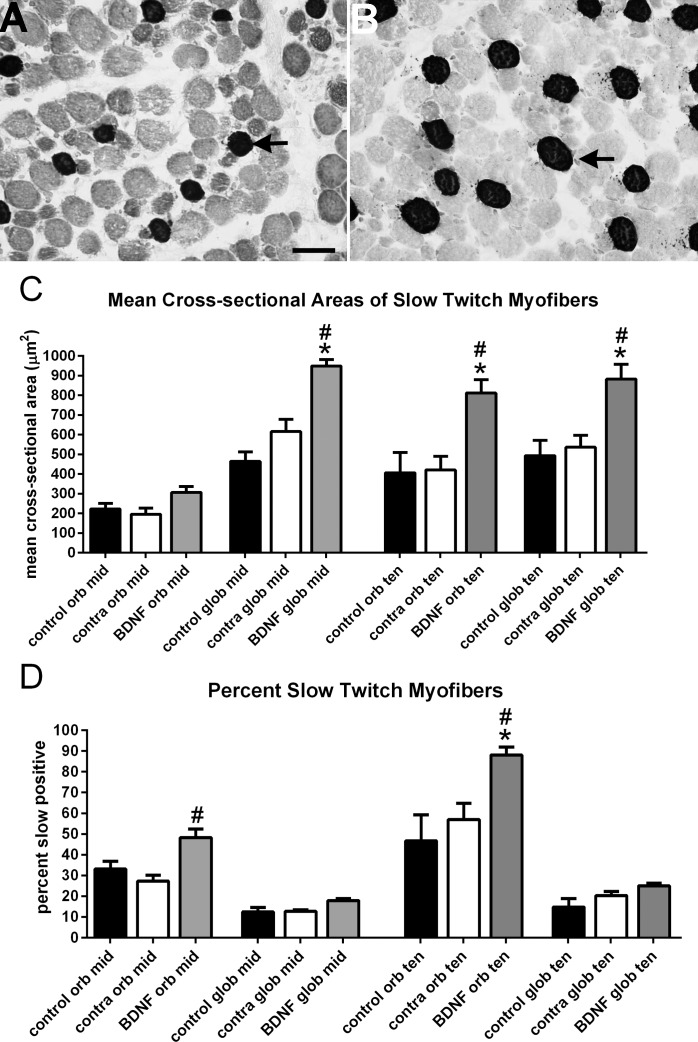
Cross-sections from the midregion global layers of (**A**) a naïve control superior rectus muscle and (**B**) a superior rectus 1 week after three serial injections with BDNF. *Arrows* indicate myofibers positive for the slow-twitch MyHC. *Scale bar*: 50 μm. (**C**) Mean cross-sectional areas of slow twitch myofibers from both the orbital and global layers from the midregion and toward the tendon region of naïve control, BDNF-treated superior rectus muscles, and from those on the side contralateral to the injection (contra). (**D**) Percent of myofibers that express the slow-twitch MyHC isoform from both the orbital and global layers from the midregion and toward the tendon region of naïve control, BDNF-treated superior rectus muscles, and from those on the side contralateral to the injection. *Significant difference from the naïve controls in the same region/layer of the muscles. #Significant difference from the muscles contralateral to the injected side in the same region/layer of the muscles.

Analyses of the mean cross-sectional areas of the myofibers that expressed the slow twitch MyHC were performed. The slow-twitch, MyHC-positive myofibers in the midregion of the superior rectus muscles 1 week after three sequential injections of BDNF showed a significant increase in mean cross-sectional area in the global layer myofibers only, with a mean of 464.7 ± 47.9 μm^2^ in the control muscles, 616.5 ± 61.45 μm^2^ in the superior rectus muscles contralateral to the treated muscles, and 949.9 ± 32.06 μm^2^ in the BDNF-treated muscles (Fig. 4A–C), a 104% increase in size compared with naïve controls and a 54.1% increase in size compared with the contralateral, untreated muscles. In the sections toward the tendon end, the mean cross-sectional areas of both the orbital and global myofibers positive for the slow-twitch MyHC isoform were significantly increased in the BDNF-treated superior rectus muscles compared with both the control and contralateral superior rectus muscles. For the orbital layer, mean cross-sectional areas of control myofibers positive for the slow-twitch MyHC isoform were 406.1 ± 103.7 μm^2^ and the contralateral muscle fibers were 421.1 ± 69.49 μm^2^ compared with a mean of 812.0 ± 67.67 μm^2^ for the BDNF treated muscles, an increase of 99% and 92.8%, respectively. For the global layer, the mean cross-sectional areas of the control slow-twitch, MyHC-positive myofibers were 493.9 ± 76.96 μm^2^ and 536.3 ± 60.27 μm^2^ for the contralateral muscles compared with 883.1 ± 74.79 μm^2^ in the BDNF-treated muscles, a 78.8% and a 64.7% increase, respectively. When the percentage of slow-twitch, MyHC-positive myofibers was examined in both the middle and tendon regions, only the orbital layer myofibers were significantly different. In the midregion, the percent of slow twitch myofibers in the control muscles was 33.1 ± 3.68% and 27.3 ± 2.9% positive in the contralateral superior rectus muscles, compared with 48.32 ± 4.1% in BDNF-treated muscles in the midregion, a 45.9% and 77% increase, respectively. In the tendon region, the orbital myofibers in control muscles were 46.69 ± 12.5% positive and 57.1% ± 7.8% positive in the contralateral muscles compared with 88.11 ± 3.8% in BDNF-treated muscles (Figs. 4A, 4B, 4D), which represented an 88.7% and 54% increase after BDNF treatment, respectively.

**Figure 5:**
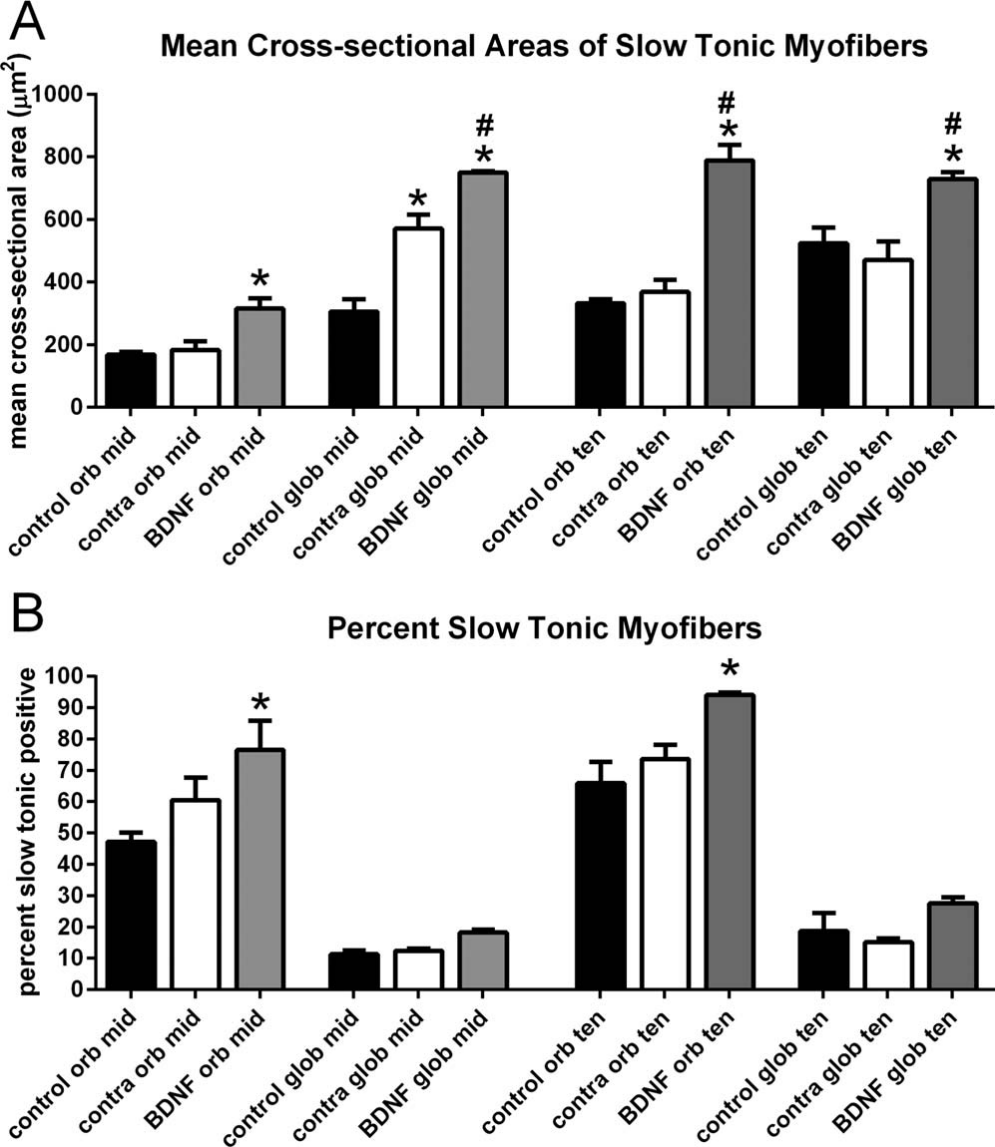
(**A**) Mean cross-sectional areas of myofibers that express the slow tonic MyHC isoform from both the orbital and global layers from the midregion and toward the tendon region of naïve control, BDNF-treated superior rectus muscles, and those on the side contralateral to the injection (contra). (**B**) Percent of myofibers that express the slow tonic MyHC isoform from both the orbital and global layers from the midregion and toward the tendon region of naïve control, BDNF-treated superior rectus muscles, and those on the side contralateral to the injection (contra). *Significant difference from the naïve controls in the same region/layer of the muscles. #Significant difference from the muscles contralateral to the injected side in the same region/layer of the muscles.

The mean cross-sectional areas and percent expression of slow tonic MyHC was examined in the three groups of superior rectus muscles (Fig. 5). There were significant increases in mean cross-sectional areas of the BDNF-treated muscles in both the orbital and global layers in the midregion, with a mean cross-sectional area in the treated orbital layer myofibers of 315.8 ± 32.7 μm^2^ compared with naïve control myofibers, with a mean of 168.6 ± 9.2 μm^2^. In the global layer, the treated myofibers had a mean cross-sectional area of 750.1 ± 3.49 μm^2^, compared with both the naïve control myofibers, with a mean of 305.4 ± 25.65 μm^2^, and the superior rectus contralateral to the treated muscles, at 571.2 ± 44.13 μm^2^ (Fig. 5A). This represented increases of 45.6% and 31.3%, respectively. In the tendon region, both the orbital and global layer myofibers of the BDNF-treated muscles were significantly larger when compared with both control and contralateral superior rectus muscle myofibers. In the orbital layer of the tendon region, the BDNF-treated myofibers were 137.2% and 113.9% larger than the control and contralateral muscles, respectively. Similarly, the BDNF-treated myofibers in the global layer were 39.17% and 55.23% larger than control and contralateral superior rectus muscle myofibers. No significant differences were seen in either the orbital or global layer myofiber cross-sectional areas between the naïve control and muscles contralateral to BDNF treatment in the tendon region. The only significant differences in the percentage of slow-tonic MyHC-positive myofibers were between the BDNF-treated orbital layer myofibers in both the midregion and tendon ends. In the orbital layer myofibers in the midregion of the muscles, the slow-tonic positive fibers in the BDNF-treated muscles were increased by 62.11%, at 76.58 ± 9.24%, compared with the naïve control muscles, at 47.24 ± 2.87%. In the orbital layer fibers of the tendon end of the muscles, the BDNF-treated myofibers were increased by 42.8% compared with the naïve controls, at 94.1 ± 0.77% compared with 65.89 ± 6.79%, respectively. Thus, within 1 week, local BDNF treatment was able to cause significant changes in both slow-twitch and slow-tonic myofiber cross-sectional areas and overall percentage of myofibers expressing these MyHC isoforms. We also examined the percentages of myofibers expressing fast, developmental, or neonatal MyHC isoforms; there were no significant differences between the BDNF-treated muscles and either control group in the expression patterns of these three MyHC isoforms (data not shown).

**Figure 6:**
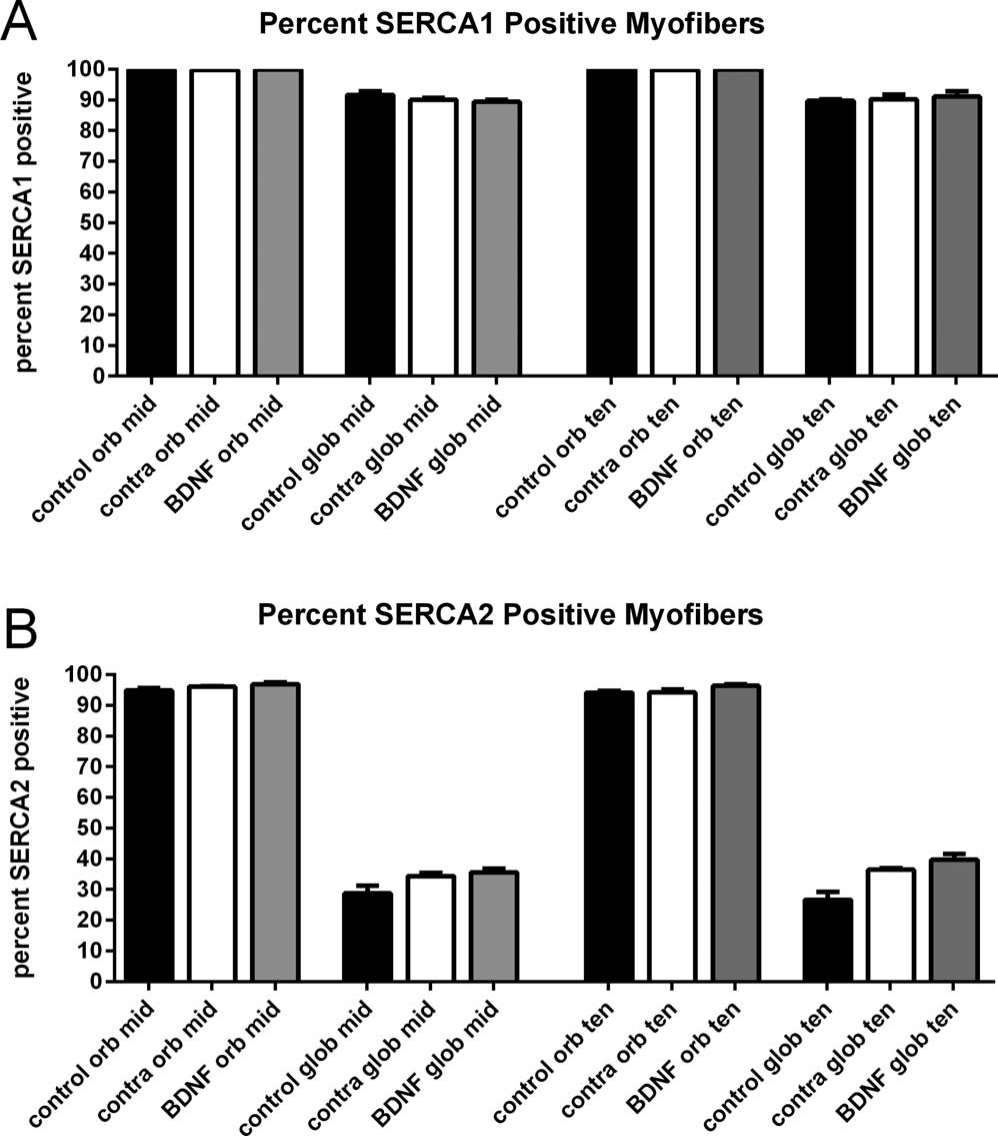
(**A**) Percent of myofibers that express SERCA1 from both the orbital and global layers from the midregion and toward the tendon region of naïve control, BDNF-treated muscles, or muscles contralateral to the treated muscles (contra). (**B**) Percent of myofibers that express SERCA2 from both the orbital and global layers from the midregion and toward the tendon region of control, BDNF-treated muscles, or muscles contralateral to the treated muscles (contra). There were no significant differences seen among any of the three groups of rabbits in the expression of either SERCA1 or SERCA2.

As the intramuscular BDNF treatment resulted in increased duration of time to relaxation, we examined the percent of myofibers that expressed SERCA1 and SERCA2 in these three groups of muscles. There were no significant differences between the percentages of myofibers positive for SERCA1 or SERCA2 between the BDNF-treated superior rectus muscles compared with either the contralateral untreated control muscles or naïve control muscles (Fig. 6).

**Figure 7:**
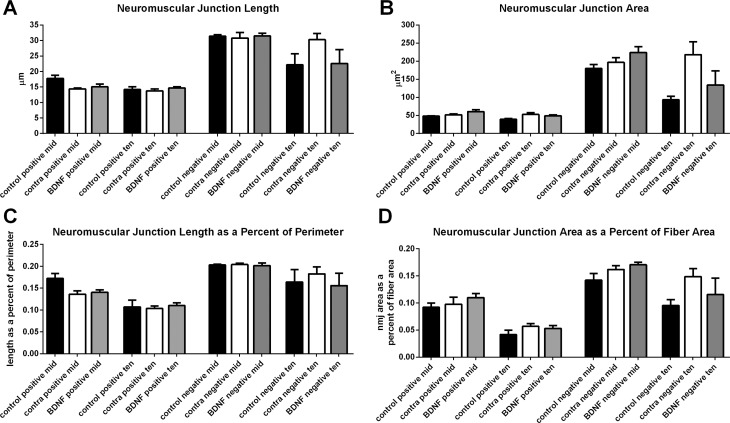
Morphometric analyses of neuromuscular junctions in naïve control, BDNF-treated muscles, and muscles contralateral to the treated muscles (contra) examined as (**A**) length, (**B**) area, (**C**) length as a percent of perimeter, and (**D**) area as a percent of myofiber area. There were no significant differences seen among any of the three groups of rabbits in any of these neuromuscular junction parameters.

Analyses of the neuromuscular junction length, area, length as a percent of myofiber perimeter, and area as a percent of myofiber area were performed for both slow MyHC-positive and slow MyHC-negative myofibers (Fig. 7). No significant differences were seen in any of the measured parameters between the three groups of superior rectus muscles analyzed.

## Discussion

Brain derived neurotrophic factor acutely administered intramuscularly to the superior rectus muscles in adult rabbits resulted in a significant prolongation of relaxation duration, with BDNF having its main effect on extending the rate at which the muscles relaxed after stimulation. It had no effect on the speed of contraction, time to peak, or on the overall force obtained. In addition, BDNF treatment resulted in increased mean cross-sectional areas in myofibers expressing the slow-twitch and slow-tonic MyHC isoforms in the tendon regions and global layer of the midregion of the muscles, and an increase in the percentage of myofibers expressing slow twitch and tonic MyHC isoforms in the orbital layer of both the middle and tendon regions.

Brain derived neurotrophic factor is normally retrogradely transported to motor neurons from the periphery,^16,17^ where it acts as a survival factor in development and after nerve injury.^3,18,19^ Brain derived neurotrophic factor and its receptors have been shown specifically to be expressed in both oculomotor neurons and extraocular muscles during development^5^ and in adults.^7^ Brain derived neurotrophic factor has a wide array of additional functions that have been identified. For example, when supplied to regenerating peripheral nerve, there was a preferential increase, and basically a return to normalcy, in the cross-sectional areas of slow MyHC-positive myofibers.^20^ The administration of BDNF in the current study showed a similar effect and resulted in increased mean cross-sectional areas and percent of myofibers expressing slow-twitch and slow-tonic MyHC. We saw this effect in another study, where continuous application of BDNF in infant monkeys resulted in an increase in both the number and mean cross-sectional areas of slow myofibers compared with that seen in age-matched controls.^6^ MyHC isoforms play a role in the control of shortening velocity^21,22^; despite changes in the proportion of myofibers expressing slow-twitch and tonic MyHC isoforms, no significant changes in shortening velocity or force were seen at this acute time-point.

In skeletal muscle, changes in SERCA isoform expression are involved in regulating fiber relaxation rate.^11^ In body skeletal muscles, SERCA1 is typically coexpressed in fast MyHC fibers while SERCA2 is coexpressed with slow MyHC isoforms.^23^ However, the EOM differ from this pattern, as the majority of myofibers were shown to coexpress both sarco(endo)plasmic reticulum Ca(2+) ATPases SERCA1 and 2 in human EOM.^24^ Interestingly, the vast majority of slow myofibers in EOM were shown to express SERCA1, but also coexpressed SERCA2 in a layer-specific manner. Our rabbit data agree with this previous study, showing that in control rabbit EOM, the vast majority of the myofibers expressed SERCA1, while only the orbital layer showed a high level of SERCA2 expression. Due to the significant coexpression patterns of the SERCA proteins in the EOM, it was not altogether unexpected that no significant changes were seen in SERCA expression after BDNF treatment.

In a previous study, the only significant effects of 3 months of sustained release of BDNF treatment to the infant monkey rectus muscles were increased mean cross-sectional areas of the slow myofibers, with proportional increases in the size of their neuromuscular junctions.^7^ There is a large literature demonstrating that BDNF plays a role in synaptic maturation in developing neuromuscular junctions^25,26^ and in synaptic plasticity, excitability, and synaptic consolidation in adults.^27,28^ However, its specificity to slow MyHC expressing myofibers in skeletal muscle has not been previously noted. It appears that even short-term administration of BDNF can produce changes to the percentage and size of slow myofibers in rabbit extraocular muscle. This finding agrees with previous work in cardiac muscle, where BDNF signaling enhanced cardiomyocyte contractility and relaxation.^29^ This result was somewhat surprising as no other MyHC isoform percentages changed; however, we have seen rapid changes in MyHC isoforms within 1-week periods after resection^30^ or recession surgery,^14^ and after IGF-I treatment.^12^ Recent studies suggest that RNA modifications allow for dynamic regulation of gene expression, and explain how rapid changes in protein synthesis can occur without requiring a change in overall levels of mRNA within cells.^31,32^

Insight into the potential functional role of retrogradely transported BDNF on the motor neurons that innervate the treated muscles comes from an elegant study by the Pastor laboratory.^10^ After cutting the abducens nerve within the orbit of adult cats, BDNF provided to the proximal stump resulted in a preferential return of the tonic phase of motor neuron firing rather than the phasic firing.^10^ In contrast, using the same experimental paradigm, neurotrophin-3 resulted in return of the phasic motor neuron firing but not the tonic firing.^10^ While the mechanism for these effects is unclear, MyHC isoform composition was altered as a result of experimental modifications to motor neuron firing patterns.^33,34^ For example, chronic low-frequency stimulation of fast muscle induced fast to slow transitions in both myosin heavy and light chains in rabbit and rat hindlimb muscle.^35,36^ One hypothesis based on the results of the present study, which requires further testing, is essentially the converse; that BDNF treatment may increase both the duration of relaxation time and mean slow myofiber cross-sectional area by altering the firing characteristics of the motor neurons.^10^

The ability to alter the relaxation profile of EOM has potential therapeutic value for the treatment of eye movement disorders, as one would predict that a longer treatment time might magnify these effects. In INS, for example, uncontrolled oscillatory movements could theoretically be at least partially decreased by treatment with BDNF or other agents with similar modulatory effects on relaxation time. In a recent study, we showed that extraocular muscles removed at the time of surgery from children with idiopathic nystagmus or nystagmus associated with albinism were significantly different from age-matched control extraocular muscles in a number of ways.^9^ Most germane to the current study was our demonstration that both groups of EOM from subjects with nystagmus had little to no expression of BDNF in the myofibers. Interestingly, these muscles also showed increased numbers and mean cross-sectional areas of slow myofibers, which we hypothesize may be compensatory. We currently are testing this hypothesis in an animal model of nystagmus. As even a short-term treatment of the rabbit EOM with BDNF was able to lengthen the time for the muscles to relax, its absence might play a role in the development and/or maintenance of the abnormal eye movements. Further studies are needed, but the data suggest that treatment of the extraocular muscles with BDNF might be able to modify and dampen the uncontrolled movements in nystagmus.

Brain derived neurotrophic factor delivered by cannula or osmotic pump to the vestibular nucleus complex has been shown to be effective in damping nystagmus following an experimental labyrinthectomy.^37^ While nystagmus induced by a labyrinthectomy is different from idiopathic forms that occur in childhood in the absence of brain or other known injury, the efficacy of BDNF in this context is very interesting. As vestibular neurons provide afferent input onto the motor neurons that directly control extraocular muscle contraction, it is possible that efficacy could be the result of anterograde transport of BDNF to the motor neurons as well as its potential effects on firing properties of vestibular nuclear neurons.

In summary, acute treatment of extraocular muscles with BDNF prolonged extraocular muscle relaxation time and rate of relaxation after stimulation at 10 and 20 Hz as well as increased the size of slow myofibers. Our previous study showing reduced/absent BDNF expression in EOM of children with nystagmus suggests that providing BDNF to the EOM may have the ability to dampen the uncontrolled oscillatory movements. Current studies are directed at answering this question.
